# Effect of *Lactobacillaceae* Probiotics on Colonic Microbiota and Metabolite Production in Cystic Fibrosis: A Comparative In Vitro Study

**DOI:** 10.3390/nu15173846

**Published:** 2023-09-03

**Authors:** Andrea Asensio-Grau, Joaquim Calvo-Lerma, Miguel Ferriz-Jordán, Jorge García-Hernández, Ana Heredia, Ana Andrés

**Affiliations:** 1 Institute of Food Engineering (IIA-FoodUPV), Polytechnic University of Valencia, Camino de Vera s/n, 46022 València, Spain; anasgr@upv.es (A.A.-G.); mferjor@upv.es (M.F.-J.); aandres@tal.upv.es (A.A.); 2 Joint Research Unit NutriCura^PDig^, Avda. Fernando Abril Martorell 106, 46026 València, Spain; 3 Advanced Food Microbiology Centre (CAMA), Polytechnic University of Valencia, Camino de Vera s/n, 46022 València, Spain

**Keywords:** gut microbiota, dysbiosis, cystic fibrosis, probiotics, short-chain fatty acids

## Abstract

Cystic Fibrosis-related gut dysbiosis (CFRGD) has become a recognised complication in children with this condition, and current evidence remains insufficient to guide the selection of probiotic strains for supplementation treatments. The aim of this study was to characterise the effect of three probiotic strains on CFRGD by means of a dynamic in vitro simulation of the colonic fermentation (SHIME^®^). The configuration of the system included three bioreactors colonised with the faecal inoculum of a child with cystic fibrosis. For 20 days, each bioreactor was supplied daily with either *Lacticaseibacillus rhamnosus GG* (ATCC 53103 TM), *Limosilactobacillus reuteri* (DSM 17938) or *Lactiplantibacillus plantarum* (DSM 22266). The baseline microbiota was characterised by a high abundance of *Prevotella*, *Faecalibacterium* and *Acidaminococcus* genera. After 20 days of supplementation, *L. rhamnosus* and *L. plantarum* reduced *Prevotella* significantly, and the three strains led to increased *Faecalibacterium* and *Bifidobacterium* and decreased *Acidaminococcus*, with some of these changes being maintained 10 days after ceasing supplementation. The metabolic activity remained unaltered in terms of short-chain fatty acids, but branched-chain fatty acids showed a significant decrease, especially with *L. plantarum*. Additionally, ammonia decreased at 20 days of supplementation, and lactate continuously increased with the three strains. The effects on colonic microbiota of *L. rhamnosus*, *L. reuteri* or *L. plantarum* were established, including increased beneficial bacteria, such as *Faecalibacterium*, and beneficial metabolites such as lactate; and on the other hand, a reduction in pathogenic genera, including *Prevotella* or *Acidaminococcus* and branched-chain fatty acids, overall supported their use as probiotics in the context of CFRGD.

## 1. Introduction

Cystic fibrosis (CF) is a life-limiting genetic disease that leads to the production of thick secretions predominantly by the lungs and pancreas, affecting the gastrointestinal tract and pulmonary function. In the case of children with CF, the consequences on growth and nutritional status are of great importance [1].

Studies suggest that dehydrated, acidic luminal environment and thick mucus within the gut [2], together with high-fat diets [3] and frequent antibiotic therapy [4], could be partly responsible of dysbiosis and intestinal inflammation reported in CF [5,6]. Compared to non-CF children, the CF microbial ecosystem is characterized by poor diversity and microbial imbalance [7,8]. Burke et al. (2013) reported that CF gut microbiota were dominated by Actinobacteria and Firmicutes, while in non-CF controls, Bacteroidota accounted for 39% of phyla, compared to 4% in the CF study group [9]. In addition, certain key bacteria were reported in several studies to have altered relative abundance in CF compared to controls, such as *Enterobacteriaceae*, including *E. coli*, *Fusobacteria*, and other species associated with the small intestine [10,11,12]. On the other hand, the relative abundance of some potentially beneficial bacteria such as *Bifidobacterium*, *Akkermansia* and butyrate producers such as *Faecalibacterium prausnitzii* and *Roseburia* are decreased in CF [5,13,14,15]. In this sense, recently, the term “cystic fibrosis-related gut dysbiosis” (CFRGD) is used [13].

Different approaches have been assessed to correct CFRGD. Among them, prebiotic, probiotic, or even vitamin D administration can be cited [16,17]. Coffey et al. (2020) gathered the trials to evaluate different probiotic strains in the CF context. In the 12 assessed human trials, a positive effect of *L. reuteri* or *L. rhamnosus* (commonly referred to as L. GG), versus placebo improved intestinal inflammation or lung function compared to placebo groups. However, no reported results on the effect of these strains on the colonic microbial profiles were reported [10].

In the recent review published by Esposito et al. (2022), authors resolved that the available data cannot be considered as sufficient to indicate that probiotics are essential elements in the CF therapy due to the scarce scientific evidence about the modulating capacity and the benefits [18]. Additionally, it is essential to note that differences among protocols, probiotic formulas, dosage, and duration of treatments limit the comparison among trials and therefore, the potential of selecting the most promising probiotics for CF.

In this sense, in vitro models of colonic fermentation could be considered a useful tool to compare the mechanism of action of different probiotics. Through these mechanistic experiments, it is possible to control and modify the pre-colonic gastrointestinal variables to administrate, simultaneously, different strains on the same CF microbiota profile (from the same donor) and to sample at different days of treatment, in order to study the changes in the relative abundance of the species and metabolites production along the administration period [17]. Among colonic fermenters, static models report an acute response in a short period of time (24–48 h), while dynamic models allow for performing longer experiments (2–4 weeks). In addition, dynamic models are closer to the in vivo intestinal environment within the bioreactors, including dynamic pH control, and duration of the stages, among others [19].

Thus, this study aimed at evaluating the potential of supplementation with *Lacticaseibacillus rhamnosus* [20], *Limosilactobacillus reuteri* [21] and *Lactiplantibacillus plantarum* [22] on improving CFRGD by means of a comparative study in an in vitro dynamic simulator of colonic fermentation.

## 2. Materials and Methods

### 2.1. Preparation of Probiotic Strains

Three probiotic strains from the former Lactobacillus genus were selected: *Lacticaseibacillus rhamnosus* GG (ATCC 53103 TM), *Limosilactobacillus reuteri* (DSM 17938) and *Lactiplantibacillus plantarum* (Lp-115; DSM 22266) from the Polytechnic University of Valencia collection. At weekly intervals, a pure culture of the different strains was inoculated into MRS broth. Cultures were harvested during the exponential growth. Then, they were centrifuged (4000× *g*, 10 min) and washed with sterile peptone water. Plate counts were performed on MRS agar of these bacterial suspensions. The concentrations were adjusted with sterile peptone water to the required concentration (10^10^ CFU/mL). The different Lactobacilli cells were kept at this concentration of in sterile peptone water until use.

### 2.2. In Vitro Colonic Fermentation Using the SHIME^®®^

The Simulator of Human Intestine Microbial Ecosystem (SHIME^®^) allows for dynamic in vitro digestion studies, reproducing specific intestinal conditions. The experiment configuration consisted of six double-jacket vessels, three of them simulating stomach + duodenum and three more simulating the proximal colon. There was one pair of stomach + duodenum and proximal colon vessels per probiotic strain. The bioreactors were continuously stirred, and the temperature was maintained at 37 °C. The system kept the anaerobiosis in the colon vessels through a daily 30 min nitrogen flow. The pH at the proximal colon vessels was maintained 5.7–5.9 using NaOH 0.1 M or HCl 0.1 M.

The three proximal colon vessels were inoculated with bacteria obtained from a faecal sample of an 8-year-old female child with a confirmed diagnosis of CF by a sweat chloride ≥ 60 mEq/L and the presence of two disease causing mutations in the CFTR gene: confirmed diagnosis of exocrine pancreatic insufficiency by faecal elastase values < 200 µg/g of faeces and treatment with pancreatic enzyme replacement therapy. The child did not present abdominal pain, infections or antibiotic treatment two months before the study. The legal guardian of the child signed informed consent after the approval from the Ethics Committee of Polytechnic University of Valencia (Ref. P09_24_11_2021) had been obtained.

Aliquots of 20 g of fresh faecal material were weighted and mixed with 100 mL of sterilised phosphate buffer (0.1 M, pH = 6.5) and homogenised with a stomacher for 10 min. The resulting mixture was poured into falcon tubes and centrifugated for 2 min at 500× *g*-force. Then, the supernatant (25 mL) was inoculated to the three colon vessels containing 500 mL of culture medium (feed). The feed (PDNM002B) was acquired from Prodigest^®^ (Ghent, Belgium) and supplied to the system three times a day to keep the bacteria alive. The feed was composed by arabinogalactan (1.2 g/L), pectin (2 g/L), xylan (0.5 g/L), glucose (0.4 g/L), yeast extract (3 g/L), peptone (1 g/L), mucin (2 g/L), L-cystein-HCl (0.5 g/L) and starch (4 g/L).

The study design consisted of four stages with a total duration of 58 days. (1) Control period: after the inoculation, the microbiota was stabilised over 14 days by the supply of the feed to allow for the adaptation of the bacteria to the specific environmental conditions at the proximal colon (pH, retention times, culture medium, etc.). (2) Baseline period: during another 14 days, the experiment followed the same conditions as in the control period. (3) Supplementation treatment: during 20 days, each of the proximal colon bioreactors was supplied with one of the probiotic strains: *L. rhamnosus*, *L. reuteri* and *L. plantarum*, respectively. The probiotic strains were daily injected into the proximal compartments assuming a concentration of 10^8^ CFU/mL at the colonic compartment. (4) Post-treatment: 10 days followed the supplementation treatment period with no probiotic administration in the same conditions as in the control and baseline periods.

Two aliquots were taken at different times at baseline (days 0 and 15), treatment (days 10 and 20) and post-treatment (days 5 and 10) periods, which were stored at −80 °C until the analyses of microbiota (day 15 of baseline; days 10 and 20 of treatment; day 10 of post-treatment) and metabolic activity (all the time points). For all the determinations, two replicates were conducted.

### 2.3. Microbiota Composition by 16S rRNA Amplicon Gene Sequencing

Microbiota composition from the proximal colon was analysed at different days during the whole experiment (t0, t15 (baseline days 0 and 15), t25, t35 (treatment days 10 and 20), and t40 and t45 (post-treatment days 5 and 10)) by 16S rRNA amplicon gene sequencing. Total DNA was extracted from the aliquots using the Stool DNA Isolation Kit from Norgen Biotek Corp^®^ (Thorold, ON, Canada), following the manufacturer’s protocol and recommendations. The final yield of the extracted DNA was determined by fluorometry (Qubit fluorometer, Invitrogen Co., Carlsbad, CA, USA). The microbiological analysis was performed by amplification with specific primers of the V3-V4 regions of the 16S rRNA using Illumina. The primers were selected from the Klindworth et al. publication [23]. The full length primer sequences, using standard IUPAC nucleotide nomenclature, to follow the protocol targeting this region were the following:

16S rDNA gene Amplicon PCR Forward Primer = 5′

TCGTCGGCAGCGTCAGATGTGTATAAGAGACAGCCTACGGGNGGCWGCAG

16S rDNA gene Amplicon PCR Reverse Primer = 5′

GTCTCGTGGGCTCGGAGATGTGTATAAGAGACAGGACTACHVGGTATCTAATCC

Amplicons were checked with a Bioanalyzer DNA 1000 chip and libraries were sequenced using a 2 × 300 bp paired-end run (MiSeq Reagent kit v3) on a MiSeq-Illumina platform (2 × 300 bp) at the FISABIO sequencing service (València, Spain).

The sequences were filtered for subsequent analysis. Filtering and quality assessment were performed at the FISABIO sequencing service using the fastp program [24] based on quality (removal of low-quality nucleotides at the 3′ end, by 10 nucleotides windows with an average quality score under 20) and length (removal of sequences with less than 50 pb). R1 and R2 from Illumina sequences were joined using the FLASH program [25] by applying default parameters. In order to analyse the bacterial community by ASVs (Amplicon Sequence Variants), the joined data were processed in DADA2 package (version 1.28.0) [26] on R-software (R version 4.3.0 (21 April 2023)). Joined reads containing undetermined nucleotides (Ns), and those which matched against the phiX genome, were removed. Reads with a maximum expected error above one were filtered (expected error calculated from the nominal definition of the quality score—∑10(−Q/10)). Exact ASVs were inferred by the DADA2 algorithm, and chimeras were removed with default parameters. Taxonomy was assigned to ASVs up to species level, with the SILVA database species train set file (version 138.1).

### 2.4. Metabolic Activity: Short-Chain Fatty Acids (SCFAs), Ammonia and Lactate

SCFAs were analysed from aliquots taken at the same time points as for microbiota analyses by gas chromatography (GC-FID), according to the protocol adopted from Tallarico-Adorno et al. (2014) [27]. Analytical calibration lines were prepared for the quantification of the volatile acids of interest: acetic acid (AA), propanoic acid (PA), butyric acid (BA), valeric acid (VA), isovaleric acid (IVA) and isobutyric acid (IBA), ranging from 0 to 30 mM. The samples (2 mL) were mixed with 5 mL of H_2_SO_4_ (9.2 M) and a small amount of NaCl was incorporated along with a spoon tip to remove any traces of water in the extract. Subsequently, 0.4 mL of the internal standard solution (52.9 mM 2-Methylhexanoic acid) and 2 mL of diethyl ether were added and vortexed for 1 min. Samples were centrifugated 3000× *g*-force for 3 min and the supernatant was transferred to the chromatography vials and injected in the equipment Agilent GC7890B-5977B GC-FID (Agilent, Santa Clara, CA, USA) with a multipurpose sampler with a SUPELCOWAX™ 10 Capillary GC Column (30 m × 0.25 mm × 0.25 μm, Merck, Rahway, NJ, USA). The oven temperature program was 90 °C for 1 min, ramped to 190 °C at a rate of 5 °C/min, and finally held at 250 °C for 30 min. Helium was used as carrier gas at a flow rate of 1 mL/min with an inlet temperature of 250 °C, and the injection volume was 2 μL. Results were expressed in millimolar concentration (mM).

The concentration of ammonia was quantified using the R-Biopharm Ammonia kit (Darmstadt, Germany) and measured in a spectrophotometer (UV/vis, Beckman Coulter, Brea, CA, USA), following the manufacturer’s instructions and recommendations. Results were expressed in micromolar concentration (mM). The lactate concentration was quantified using the Lactate Assay kit of Sigma Aldrich^®^ (St. Louis, MO, USA, EE. UU) using a Multiskan FC Microplate reader (ThermoScientific 51119100, Waltham, MA, USA) following the manufacturer’s instructions. Results were expressed in micromolar concentration (µM).

### 2.5. Statistical Analysis

Relative abundance of phylum and genera and the production of ammonia, lactate and short-chain fatty acids were measured in each pair of replicates and summarised as mean and standard deviation. Stratigraphic Centurion was used for the statistical analysis. ANOVA were applied to study the differences in the relative abundance at phylum and genus levels between different stages (control, treatment (after 10 and 20 days of supplementation) and post-treatment) and between the three probiotic strains within each stage. The difference in relative abundance at genus level between the experiment stages was expressed as the fold change. In case of the metabolic activity, ANOVA were applied to assess the differences between all the timepoints with available aliquots in all the stages: 0, 10, 15, 25, 35, 40 and 45 days. Pearson correlations were used to assess the possible relations between the microbial genera and the production of metabolites.

## 3. Results

### 3.1. Effect of Probiotic Administration on Colonic Microbiota Populations

**Figure 1:**
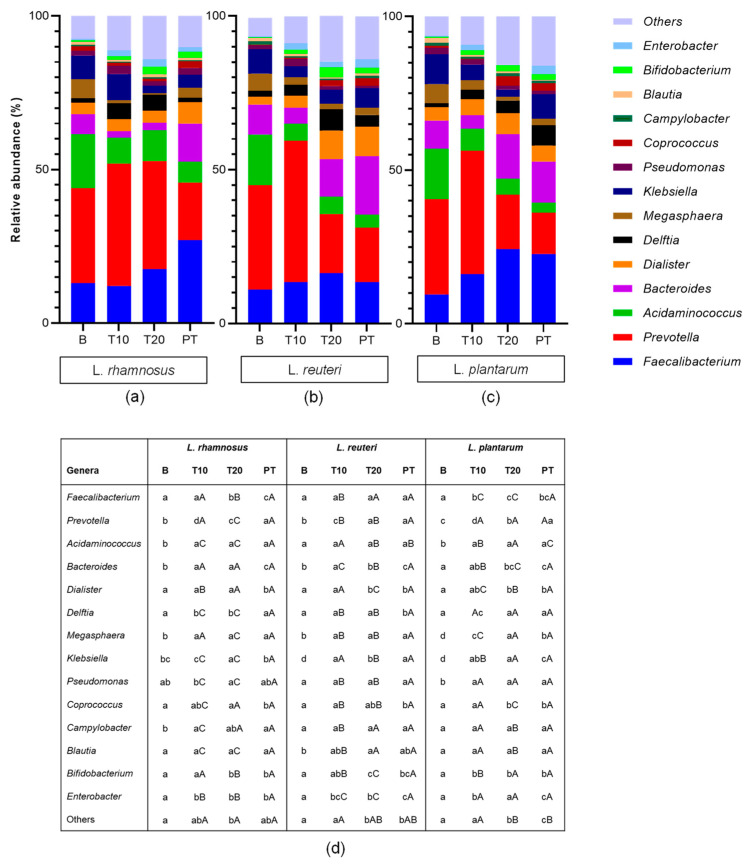
Relative abundance of bacteria at genera taxonomic level in the colonic environments supplemented with (**a**) *L. rhamnosus*, (**b**) *L. reuteri* and (**c**) *L. plantarum* at four time points of the experiment: baseline (B), 10 days of supplementation treatment (T10), 20 days of supplementation treatment (T20) and 10 days of post-treatment (PT). (**d**) statistically significant differences between study periods (lower case letters) and probiotic strains (capital letters).

The changes in colonic microbiota induced by the three study strains throughout the experiment periods, are presented in Figure 1. At the experiment onset (baseline period), the relative abundance range among the three bioreactors showed that *Faecalibacterium* (9.5–12.9%) and *Prevotella* (30.9–33.9%) were the most abundant bacterial genera. *Acidaminococcus* was the third most abundant genus (16.4–17.6%), followed by *Bacteroides* (6.4–9.7%) *Klebsiella* (7.7–9.7%) and *Megasphaera* (5.7–6.3%). In contrast, *Bifidobacterium* genus was found in relatively low abundance (0.31–0.56%), while *Enterobacter* was found in 0.24–0.42%. No statistical differences among the three bioreactors were found at the baseline period.

After 20 days of supplementation treatment, nine bacteria genera showed statistically significant changes in the relative abundance compared to baseline, with variable extents depending on the supplied probiotic strain. *Faecalibacterium* genus presented a significant increase in all three treatments, which corresponded with a fold change of 1.42 with *L. rhamnosus* supplement, 1.3 for *L. reuteri*, and 2.64 *L. plantarum* (*p* < 0.05). In addition, an increase in *Bifidobacterium* was detected with fold changes of 4.5–5.5, which were statistically significant in all the cases. Inversely, *Prevotella* was significantly reduced (*p* < 0.05) but only in the colonic environments treated with *L. reuteri* (fold change 0.55) and *L. plantarum* (fold change 0.58). *Acidaminococcus* also accounted for a significant decrease as the consequence of the three probiotic strains’ supplementation; notably, the *L. plantarum* strain contributing the most to the reduction (0.4). Regardless of the strain, the probiotic treatment also affected *Megasphera*, *Klebsiella* and *Campylobacter*, decreasing their presence on microbiota. Regarding *Enterobacter*, an increase with *L. rhamnosus* and *L. reuteri* was detected, but *L. plantarum* led to the suppression (0.000%) of this genus. In turn, *Bacteroides* decreased when *L. rhamnosus* was administrated.

Focusing on the post-treatment period, the changes achieved during the supplementation treatment for some bacterial genera were maintained, while for others, the tendency was returning towards baseline values. Concretely, *Faecalibacterium* abundance was maintained or even increased in the case of *L. rhamnosus* and *L. plantarum* treatments, but dropped to baseline values for *L. reuteri*. All three strains were able to maintain the reduced values of *Prevotella* and *Acidaminococcus*. As for *Bacteroides*, the relative abundance was maintained with *L. reuteri* and *L. plantarum* at comparable levels to those detected at the end of the supplementation treatment period. In turn, *L. rhamnosus* led to an increase in the relative abundance of this genera to comparable values of the other two probiotic strains. However, the levels of *Bifidobacterium* were significantly reduced compared to the 20-day treatment period.

The results in colonic microbiota at the phylum level are available in Figure S1. At this taxonomic level, the three strains could reduce Proteobacteria along with increasing Firmicutes, but decreasing Bacteroidota after 20 days of supplementation.

### 3.2. Effect of Probiotic Administration on Metabolic Activity

**Figure 2:**
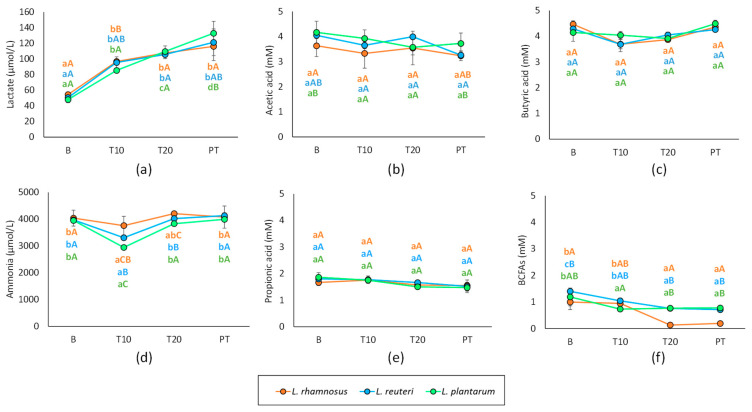
Metabolic activity products of colonic microbiota over the study stages and treatments with *L. rhamnosus*, *L. reuteri* and *L. plantarum*. (**a**) lactate, (**b**) acetic acid (**c**) butyric acid, (**d**) ammonia, (**e**) propionic acid, (**f**) sum of branched chain fatty acids, BCFA, including isobutyric acid, IBA, and isovaleric acid, IVA. A–C letters refer to the homogenous groups obtained for different probiotic; a–b letters indicate the homogeneous groups in terms of experiment stage.

The three probiotic strains had an impact on ammonia and lactate production (Figure 2). Concretely, ammonia concentration depicted a significant decrease at 10 days of supplementation treatment with the three probiotic strains (3755 mM *L. rhamnosus*, 3305 mM *L. reuteri* and 2943 mM *L. plantarum*), but returned to basal values at the timepoint of 20 days (close to 4000 mM) and was maintained during the post-treatment period as well. In turn, lactate production showed an increasing tendency after the supplementation treatment onset, which allowed for incrementing the baseline values of 48.1–54.6 mM to 105.8–109.6 mM at 20 days of treatment. The production also kept increasing during the post-treatment period to a maximum of 116.2–133.0 mM. When assessing differences depending on the type of probiotic strain, *L. plantarum* showed the highest decrease in ammonia at 20 days of treatment (*p* < 0.05), while the increased lactate production was not affected by the type of strain regardless of the study timepoint (*p* > 0.05).

As for the other metabolites, the SCFA, the concentration of AA, PA and BA were found to be the majoritarian with mean values ranging from 3.5 to 4.5 mM in AA and BA, and close to 2 mM in PA throughout the experiment. (Figure 2). The results showed no significant changes over the experiment stages. However, the BCFA, which were in a lower concentration than AA, BA and PA, were significantly reduced after the treatment, with post-treatment of *L. plantarum* as the one reducing BCFA the most.

### 3.3. Correlation between Gut Microbiota and Metabolic Activity

**Figure 3:**
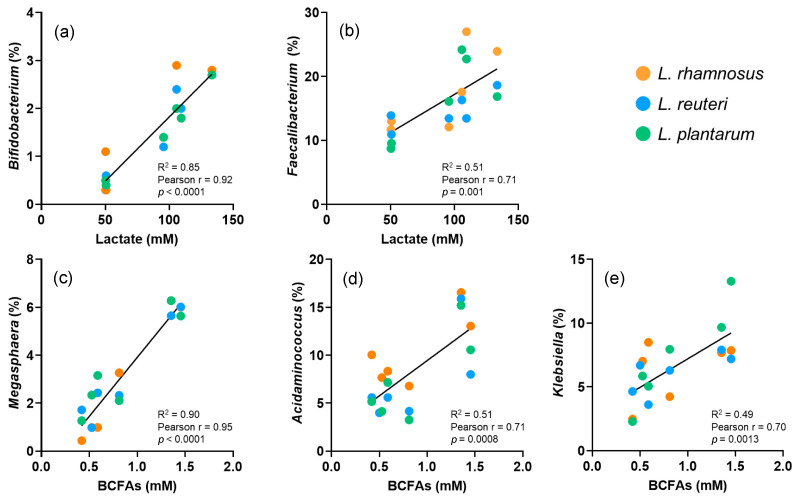
Statistically significant correlations (Pearson) between the relative abundance of bacterial genera and the concentration of metabolite products considering all the stages of the study. (**a**) *Bifidobacterium* vs. lactate. (**b**) *Faecalibacterium* vs. lactate. (**c**) *Megasphaera* vs. BCFAs. (**d**) *Acidaminococcus* vs. BCFAs. (**e**) *Klebsiella* vs. BCFAs. BCFAs, branched chain fatty acids.

The relative abundance of some bacterial genera showed some significant correlations with the metabolites’ production (Figure 3). The genera *Bifidobacterium* and *Faecalibacterium* were positively and significantly correlated with the production of lactate (R^2^ = 0.85, *p* < 0.0001; and R^2^ = 0.51, *p* = 0.001, respectively). In contrast, *Megasphaera*, *Acidaminococcus* and *Klebsiella* showed a positive and significant correlation with the production of BCFA (R^2^ = 0.90, *p* < 0.0001; R^2^ = 0.51, *p* = 0.0008; and R^2^ = 0.49, *p* = 0.0013, respectively).

## 4. Discussion

This study evaluated the potential of three probiotic strains (*Lacticaseibacillus rhamnosus* GG (ATCC 53103 TM), *Limosilactobacillus reuteri* (DSM 17938) and *Lactiplantibacillus plantarum* (DSM 22266)) for improving the colonic microbiota in the CF context. The findings point to a positive effect in terms of change in the relative abundance of relevant bacterial genera and metabolite production. Although similar effects were accounted by the three study strains, some showed specific effects in the assessed parameters.

The first relevant finding is the reduced abundance of *Prevotella*, which was the majoritarian genus in the baseline microbiota. It was previously described that the gut ecosystem is prone to entail *Prevotella* or *Bacteroides*, but not both, as in the present study [28]. However, in one of the few studies on colonic microbiota in children with CF, only 1/24 of subjects showed *Prevotella* as the majoritarian genera [10]. More than 50 species of *Prevotella* are known, some of them associated with plant-based diets or with inflammation processes, while others are common in infections of the oral cavity [29]. Due to the altered intestinal conditions of children with CF [30], the predominance of *Prevotella* species in CF donor’s faecal inoculum could be related to gut inflammation [31], which contributes to colonic dysbiosis [32]. However, the available studies on the role of *Prevotella*, do not describe its effects in CF. Altogether, our findings suggest that the treatment with *L. reuteri* and *L. plantarum* (but not *L. rhamnosus*) could be a useful approach to reduce *Prevotella* after 20 days of supplementation in children with CF affected by this type of dysbiosis. The reason for excluding *L. rhamnosus* is that it was unable to achieve a significant reduction in *Prevotella*. It is difficult to explain this difference with respect to the other strains, but a recent study also found different effects of *L. reuteri* vs. *L. rhamnosus* on gut microbiota in mice [33]. Notably, *L. rhamnosus* was not only unable to decrease *Prevotella*, but also contributed to decrease *Bacteroides* at the end of the supplementation treatment (unlike the other two strains), reinforcing the premise that these two genera tend to present inverse proportions [28].

Probiotic supplementation, regardless of the strain, was also effective in reducing *Acidaminococcus*. This genus was identified as specific in CFRGD, correlated with inflammation [10,34,35], and associated with lower growth rates in the CF young population [36]. The supplementation with the three strains did also significantly reduce the abundance of *Megasphera*, a CF-specific and pathogenic-related genus [10,34], and also the genus *Klebsiella* from which some species are related to lung infection in CF [36], and are considered as additional positive results. In contrast, the supplementation with *L. rhamnosus* and *L. reuteri* led to an increase of *Enterobacter* during treatment, which is considered a negative result. However, despite the statistically significant change, it may not be biologically relevant, as it increased <2% in relative abundance, and the other reported positive effects could compensate this drawback.

Furthermore, the supplementation treatment was also effective in increasing the abundance of beneficial-related bacteria, i.e., *Bifidobacterium* and *Faecalibacterium*. These two genera are known to be producers of lactate, which are related to beneficial health outcomes [37,38]. In fact, the correlation analysis between these two genera and the production of the cited metabolite showed a positive and significant tendency. Moving to the produced SCFA, no significant changes were found, suggesting that the variations in colonic microbiota populations did not modify their ability to metabolise the fermentable compounds (arabinogalactan, pectin, xylan) present in the feed medium. It is documented that a wide range of bacterial genera are able to produce SCFA, so in spite of changes in the relative abundance of different bacterial genera, the SCFA production remained unaltered [39]. In fact, previous studies on the effect of probiotics supplementation did not report changes in SCFA production either [40]. However, an exception was registered regarding the branched-chain species. These metabolites are produced through the breakdown of protein-based molecules [41], such as peptone and L-cysteine contained in the feed, although other studies also suggest that when dead cells are broken down, they produce BCFA [40]. BCFA are related to the proteolytic activity that results in the production of potentially adverse compounds such as amines, phenols, and sulphides [42]. Thus, the changes in colonic microbiota imparted through probiotic supplementation with *L. Plantarum*, the strain reducing BCFA the most, suggest a suppression of those species with the ability to metabolise protein. Concretely, according to the correlation analysis, these species were *Megasphera*, *Acidaminococcus* and *Klebsiella*, with previous records on their ability to produce BCFA [43]. To sum up, the metabolic profile of the microbiota was enhanced, or at least, not exacerbated, with the supplementation of the three probiotic strains, and the improvement was related to changes in the abundance of specific beneficial or pathogenic bacterial genera.

It is important to point out that the significance of this study is restricted to the in vitro scenario and its inherent limitations. However, results may contribute to increase the scarce evidence available in the scientific literature regarding the role of supplementation with *Lactobacillus*, or other probiotics, in improving CFRGD. In 2007, a study with 19 children with CF showed that *L. rhamnosus* GG improved weight gain and pulmonary function after 6 months of the administration. The study did not assess the changes in colonic microbiota or metabolic activity, and could only assume that inflammation in the lungs could be related with inflammation in the gut [44]. More recently, daily *L. rhamnosus* GG probiotic supplementation was tested in children with CF over a 12-month period, results showing that the supplementation that was induced increased *Bifidobacterium* as a dominant genus, and this was related to improved study outcomes [45]. However, the study did not assess the full range of bacterial populations and was only focused on a reduced number of genera, and the metabolic activity was not assessed either. In turn, other studies, including pilot studies, randomized and non-randomized control trials, were able to depict improvement in intestinal inflammation after supplementation with probiotics, including *Lactobacillus* strains. In this sense, some studies showed improved faecal calprotectin levels with *L. rhamnosus* GG supplementation [44,46]. To our knowledge, only one study assessed changes in colonic microbiota in children with CF induced by *L. reuteri* supplementation, showing positive results, including increased Firmicutes and Bacteroidota and decreased Proteobacteria [46], which seems in accordance with the findings of the present study. Conversely, other clinical studies in CF on the potential of *L. rhamnosus* GG on pulmonary function-related outcomes showed to have no effect, but no assessment of colonic microbiota was made [47]. In addition, none of the commented studies assessed the outcomes after ceasing the supplementation with the probiotic. On the other hand, systematic reviews have repeatedly concluded that the available studies on the role of probiotic supplementation in CF do not allow for establishing a solid criterion on the recommendation [48], and more recently, encourage that supplementation with probiotics should be made according to patients’ individual characteristics (microbiota, diet, etc.) [17]. Overall, current evidence encourages conducting new screening studies on the role of probiotics in modifying basal colonic microbiota, before addressing clinical studies with patients.

Finally, three methodological limitations are acknowledged; only one faecal sample was used as inoculum, no biological replicates were considered and no blank bioreactor was included in the experimental design. However, these limitations are present in most of the studies conducted with the SHIME^®®^ [49,50,51].

## 5. Conclusions

In summary, the message from this study is that depending on the target (e.g., reducing a specific genus or modifying metabolic activity of the microbiota), the selection of a probiotic strain should be made, which coincides with current knowledge [4]. In this sense and at least in the context of the present study, *L. rahmnosus*, the species that has been most evaluated as a probiotic supplement, is not the most effective in improving all the microbiota change indicators, such as the effect on *Prevotella* or *Enterobacter*, for which *L. plantarum* strain showed the best results. In addition, *L. plantarum* would be the most recommendable for reducing the production of BCFAs. Comparing the overall results of the three assessed strains, *L. reuteri* showed no special improvement compared with either *L. rhamnosus* or *L. plantarum*, at least, in the context of the assessed basal colonic microbiota of this study. In addition, the results encourage the follow-up of changes in microbiota and metabolic activity during the post-supplementation period.

In conclusion, this study has characterised the beneficial effects of using *L. rhamnosus*, *L. reuteri* or *L. plantarum* strains as probiotic supplements for modifying CFRGD in an in vitro comparative study (same concentration and dosing time on the CF microbiota), and provides a rationale for using them in future clinical trials.

## Data Availability

Data will be made available on request. The Illumina sequencing raw data was uploaded to the NCBI database.

## Supplementary Materials

The following supporting information can be downloaded at: https://www.mdpi.com/article/10.3390/nu15173846/s1, Figure S1. Relative abundance of bacteria at phylum taxonomic level in the colonic environments supplemented with *L. rhamnosus*, *L. reuteri* and *L. plantarum* at four time points of the experiment: (a) baseline. (b) 10 days of supplementation treatment. (c) 20 days of supplementation treatment. (d) 10 days of post-treatment.
