# Supplementary figures and images for: Effect of Lactobacillaceae Probiotics on Colonic Microbiota and Metabolite Production in Cystic Fibrosis: A Comparative In Vitro Study
Source: Nutrients. 2023 Sep 3;15(17):3846. doi: 10.3390/nu15173846 (PMC10490339; doi:10.3390/nu15173846)

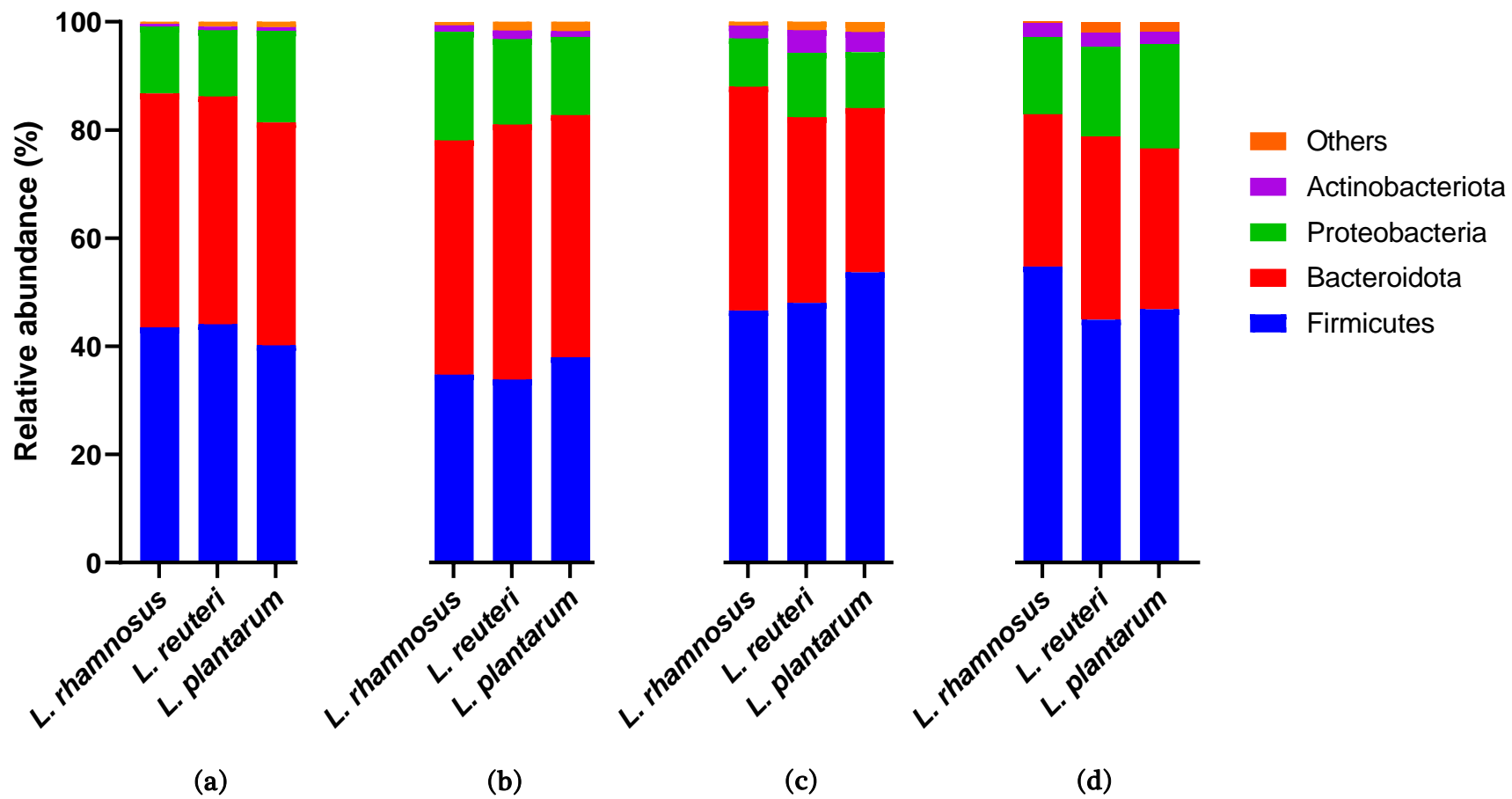

Supplement: Supplementary file 1 [file nutrients-15-03846-s001.zip › nutrients-2555184-supplementary.pdf]
